# Barriers to Safe Opioid Prescribing in Al-Ahsa Hospitals: A Multicenter Qualitative Study

**DOI:** 10.3390/healthcare14213806

**Published:** 2026-11-07

**Authors:** Rashed M. Almuqbil, Mostafa Shaban, Sayed Ibrahim Ali

**Affiliations:** 1 Department of Pharmaceutical Sciences, College of Clinical Pharmacy, King Faisal University, Al-Ahsa 36362, Saudi Arabia; ralmuqbil@kfu.edu.sa; 2 Gerontological Nursing Department, Faculty of Nursing, Cairo University, Cairo 11562, Egypt; 3 Department of Family and Community Medicine, College of Medicine, King Faisal University, Al-Ahsa 31982, Saudi Arabia

**Keywords:** opioid stewardship, safe opioid prescribing, dosage forms, route of administration, substance use disorder prevention, healthcare management, intravenous-to-oral conversion, implementation science, qualitative research, Saudi Arabia

## Abstract

**Highlights:**

**What are the main findings?**

• Across four tertiary hospitals in one Saudi region, participants described controlled-drug governance that emphasized custody and traceability and reported that none of the hospitals had a formally chartered opioid stewardship program.
• Participant-reported barriers included parenteral defaults, unstandardized opioid conversion, high-risk dosage forms without system safeguards, stigma and absent opioid use disorder pathways, and constrained pharmacist and nursing roles.

**What are the implications of the main findings?**

• Participants identified formal governance, electronic health record defaults with clinical safeguards, pharmacist-supported intravenous-to-oral conversion, standardized conversion and patient-controlled analgesia safeguards, and audit and feedback as potentially actionable hospital-level strategies that warrant prospective evaluation.
• Linking opioid stewardship to addiction services and to national accreditation standards was proposed as a policy direction; its feasibility and its effects on substance use disorder prevention and on access to analgesia require evaluation.

**Abstract:**

**Background/Objectives:** Opioid stewardship is an organizational approach to reducing opioid-related harm and iatrogenic opioid use disorder, yet implementation evidence outside North America and Europe is limited, and optimization of dosage forms and routes is rarely treated as an explicit stewardship target. This study explored stakeholders’ perceptions of barriers to safe opioid prescribing and to the optimization of opioid dosage forms and routes of administration, and the management and policy levers they considered necessary for implementing opioid stewardship, in tertiary hospitals in the Al-Ahsa region of Saudi Arabia. **Methods:** A multicenter qualitative descriptive study was conducted in four tertiary hospitals (governmental, military, and private) in Al-Ahsa, Saudi Arabia, between November 2025 and February 2026. Thirty purposively sampled stakeholders (11 physicians, 8 clinical pharmacists, 8 nurses, and 3 governance leads) completed semi-structured interviews informed by the updated Consolidated Framework for Implementation Research (CFIR 2.0). Data were analyzed using reflexive thematic analysis and reported in accordance with SRQR and COREQ; participant-proposed solutions were mapped by the authors to Expert Recommendations for Implementing Change (ERIC) strategies. **Results:** Participants across sites described controlled-drug governance that prioritized custody and traceability and reported that no site had a formally chartered opioid stewardship program. Five themes were generated: (1) “rules without a steward”, reflecting compliance-focused governance without accountable stewardship ownership; (2) “the needle is the treatment”, reflecting parenteral defaults, delayed intravenous-to-oral step-down, and formulary gaps; (3) “calculating in the dark”, reflecting unstandardized equianalgesic conversion and unsafe use of high-risk dosage forms; (4) “caught between two fears”, reflecting opiophobia, stigma, and absent addiction-care pathways; and (5) “pharmacists at the door, not at the table”, reflecting professional-boundary and workforce constraints. **Conclusions:** In these four hospitals, participants perceived a gap between the custody of controlled drugs and the clinical optimization of opioid use. Participant-identified strategies, including accountable stewardship governance, oral-route defaults with safeguards that preserve individualized and timely analgesia, pharmacist-supported conversion, audit and feedback, and linkage to addiction services, are potentially actionable but warrant prospective implementation and evaluation before effects on opioid safety or on the prevention of opioid use disorder can be inferred.

## 1. Introduction

Opioids remain indispensable for the relief of acute, procedural, and cancer-related pain, yet their benefits are inseparable from serious risks, including respiratory depression, medication errors, diversion, and the transition from therapeutic exposure to long-term use and opioid use disorder (OUD) [1,2]. Hospitals are a frequent point of first exposure: new persistent opioid use follows approximately 6% of minor and major surgical procedures [3], and the probability of long-term use rises steeply with the duration and size of the initial supply [4]. The North American opioid crisis demonstrated how prescribing norms, aggressive promotion, and weak regulatory oversight can translate individual clinical decisions into population-level harm [5]. In response, health systems have increasingly shifted attention from individual prescriber behavior toward organizational structures, including guideline-concordant prescribing [6,7,8], prescription drug monitoring programs [9], and electronic health record (EHR) defaults that shape what clinicians order [10,11,12].

Opioid stewardship has emerged as the organizing framework for these efforts. Modeled on antimicrobial stewardship, it describes coordinated, institution-level interventions to improve, monitor, and evaluate opioid use so that effective analgesia is balanced against the prevention of harm [13]. Typical components include governance by an interprofessional committee, prescribing guidance, pharmacist review, monitoring for opioid-induced sedation, naloxone co-prescribing, education, and performance metrics [14,15,16,17]. Because hospital prescribing initiates many long-term opioid trajectories, stewardship is also an upstream lever for preventing iatrogenic OUD, which places it squarely within the healthcare management and policy agenda for substance use disorder (SUD) care. Nevertheless, surveys show that opioid stewardship programs (OSPs) vary widely in structure, staffing, and scope [14], and the few implementation studies available identify leadership endorsement, workflow integration, role clarity, workload, and resourcing as decisive determinants of whether programs are sustained [18,19,20].

One stewardship dimension has received comparatively little attention: the optimization of opioid dosage forms and routes of administration. Route selection influences onset and peak effect, the likelihood of administration errors, the need for monitoring, cost, and misuse potential. In patients able to take oral medicines, oral opioids can provide analgesia comparable to intravenous patient-controlled analgesia (PCA) with lower total opioid consumption [21], and structured intravenous-to-oral switch programs, well established for antimicrobials, reduce costs and line-related harms while being hampered chiefly by clinicians’ beliefs about oral bioavailability [22]. Conversions between agents and routes are inherently error-prone: equianalgesic tables rest on limited evidence, and clinicians’ calculations for identical regimens vary widely [23,24,25,26,27]. Specific dosage forms carry distinctive hazards, including the initiation of transdermal fentanyl in opioid-naïve patients [28] and PCA programming errors [29], whereas abuse-deterrent formulations reduce non-oral tampering but do not prevent oral overuse and may displace misuse to other opioids [30,31]. Dosage-form and route decisions are therefore stewardship decisions, yet they are seldom treated as explicit program targets.

These questions are particularly salient in Saudi Arabia. Globally, access to opioid analgesics remains profoundly unequal [32,33], and in 2019 Saudi Arabia recorded the highest pharmaceutical opioid consumption in the World Health Organization Eastern Mediterranean Region [34]. At the same time, Saudi studies document opiophobic attitudes and knowledge deficits among clinicians and trainees [35,36,37,38], variable prescribing patterns [39,40,41], and organizational barriers to pain management [42]. Controlled drugs are governed by the Law of Combating Narcotic Drugs and Psychotropic Substances [43], tracked through the Saudi Food and Drug Authority’s electronic Controlled Drugs System [44], and, since 2025, prescribed through a national electronic platform for narcotic and controlled medicines [45], while hospital quality is overseen through national accreditation standards [46]. Although computerized prescriber order entry and antimicrobial stewardship are now common in Gulf hospitals, pharmacists’ clinical roles within stewardship remain limited [47]. The evidence base on SUD in the Kingdom is small and methodologically weak [48], patterns of substance use are shifting [49], and OUD treatment has historically been abstinence-based, with limited access to medication for OUD (MOUD) across the Arab region [50,51]. The Al-Ahsa region adds a further dimension: it has the highest prevalence of sickle cell disease (SCD) in the Kingdom [52,53], so recurrent vaso-occlusive crises generate substantial opioid demand. Delayed first analgesia and continued use of the intramuscular route have been documented in an Al-Ahsa emergency department [54], dosing in Saudi emergency care is frequently inappropriate [55,56], and international evidence shows that patients with SCD face stigma when seeking opioid analgesia [57,58].

To our knowledge, no published qualitative study has examined the organizational conditions for implementing opioid stewardship in Saudi hospitals or how stakeholders perceive the optimization of opioid dosage forms and routes; existing Saudi evidence consists mainly of surveys of knowledge and attitudes, analyses of prescribing patterns, and studies of barriers to pain management [35,36,37,38,39,40,41,42]. Understanding these processes in a specific regional context is a first step toward designing stewardship models that fit the Saudi regulatory and service context and to identify the management and policy levers through which health systems can prevent opioid-related harm and iatrogenic OUD. This study therefore aimed to explore, from the perspectives of physicians, clinical pharmacists, nurses, and governance leads in tertiary hospitals in the Al-Ahsa region, (1) barriers to safe opioid prescribing; (2) barriers to optimizing opioid dosage forms and routes of administration within current stewardship-related activities; and (3) organizational and policy levers that stakeholders consider necessary to implement and strengthen opioid stewardship. Because this study was confined to one region, it was intended to generate context-rich, transferable insights rather than findings representative of Saudi healthcare organizations as a whole.

## 2. Materials and Methods

### 2.1. Study Design

A multicenter qualitative descriptive design [59] was used, with semi-structured individual interviews. Qualitative description was chosen because this study sought a comprehensive, practice-oriented account of how stewardship processes operate, expressed close to participants’ own language, rather than an abstract theory. The updated Consolidated Framework for Implementation Research (CFIR 2.0) [60] served as a sensitizing framework for the interview guide and as an interpretive lens during the later phases of analysis, which allowed findings to be linked to recognized implementation determinants and, in turn, to discrete implementation strategies [61]. Reporting follows the Standards for Reporting Qualitative Research (SRQR) [62], and the Consolidated Criteria for Reporting Qualitative Research (COREQ) checklist [63] is provided in Supplementary File S1. Because data were generated through interviews, the findings represent participants’ perceptions and reported practices; no documentary audit of policies, formularies, electronic health record (EHR) configurations, or prescribing records was undertaken.

### 2.2. Setting

This study was conducted in four tertiary (referral-level) hospitals (H1–H4) in the Al-Ahsa region of the Eastern Province, Saudi Arabia. To protect participant confidentiality, sites are identified by codes. The sites were selected to represent the main governance sectors that deliver tertiary care in the region: two Ministry of Health hospitals, one military hospital, and one private hospital (H3). All sites hold national accreditation, operate electronic prescribing, and dispense controlled medicines under the national regulatory framework [43,44]. According to site coordinators and participants, including the governance leads (a documentary review was not undertaken), at the time of data collection, none of the sites had a formally chartered opioid stewardship program with dedicated staffing; opioid-related activities were distributed across pharmacy and therapeutics committees, medication safety committees, acute pain services, and palliative care teams. All four hospitals provide emergency, surgical, oncology, and hematology services, including care for adults with SCD, the dominant source of recurrent inpatient opioid use in the region [52,54].

### 2.3. Participants and Sampling

Purposive, maximum-variation sampling [64] was used to capture the perspectives of the professional groups involved across the opioid medication-use process: prescribing, verification and dispensing, administration and monitoring, and governance. Eligible participants were (a) licensed physicians, clinical pharmacists, or registered nurses with at least one year of experience at the current hospital and regular involvement in prescribing, verifying, administering, or monitoring opioids; or (b) managers or committee members with responsibility for medication safety, controlled-drug governance, or pharmacy and therapeutics decisions. Interns, residents in their first postgraduate year, and staff on extended leave were excluded. Within each site, variation was sought by specialty (emergency medicine, anesthesia and acute pain, general surgery, oncology and palliative care, hematology, and internal medicine), nationality, gender, and years of experience.

Sample size was guided by the concept of information power [65] rather than by saturation. This study aim was moderately broad, but participants held highly specific experience of the phenomenon, an established framework informed the guide, interview dialogue was expected to be strong because interviewers shared clinical backgrounds with participants, and analysis was cross-case. An initial target of 24 to 32 participants was set, distributed across the four sites and four stakeholder groups. Recruitment continued until the research team judged that the dataset held sufficient information power to answer the research questions with depth and variation across sites and roles; empirical work suggests that most codes in relatively homogeneous interview samples are identified early [66]. Adequacy was judged qualitatively rather than by the number of interviews. After each block of four to six interviews, the interviewers and analysts reviewed field notes and preliminary codes and asked whether (a) each stakeholder group and site was represented by accounts with sufficient depth to address each research question; (b) each barrier area covered by the guide (governance, route and dosage-form decisions, conversion, high-risk products, transitions of care, stigma and addiction care, and professional roles) was illuminated by detailed, concrete examples from more than one site and professional group; and (c) later interviews refined, rather than reoriented, the developing analysis. Recruitment ended when the team judged that these conditions had been met [65].

At each site, a site coordinator who was not involved in data collection or analysis circulated the same invitation and information sheet via institutional email to staff in the target departments and committees, followed by a reminder two weeks later. The three governance leads were invited directly by email from the principal investigator (R.M.A.) because of their specific roles. Interested staff contacted the research team directly, so coordinators did not know who participated. Interviewers confirmed eligibility and, when more volunteers from a professional group at a site were available than needed, prioritized those who added variation in specialty, experience, gender, and nationality. Of 37 professionals who expressed interest, 30 were interviewed; 7 did not proceed because of workload or scheduling constraints (n = 5) or a change of mind (n = 2). No participant withdrew after the interview. Because invitations were distributed through institutional mailing lists, the number of eligible staff who received the invitation and chose not to respond could not be determined. The same recruitment procedure, eligibility criteria, information sheet, consent form, and interview guide were used at all four sites.

### 2.4. Data Collection

Semi-structured interviews were conducted between November 2025 and February 2026. The interview guide was developed by the research team from the opioid stewardship and safe-prescribing literature [8,13,14] and from the five CFIR 2.0 domains (innovation, outer setting, inner setting, individuals, and implementation process) [60]. It contained role-specific questions on current opioid practices; perceived barriers to safe prescribing; decisions about dosage forms and routes, including parenteral-to-oral step-down, equianalgesic conversion, extended-release and transdermal products, and PCA; organizational structures and data; patients with SCD or SUD; and recommended improvements. All instruments, including a brief demographic form, were developed by the authors for this study, and no copyrighted or licensed questionnaire was used. The guide was piloted with two clinicians who were not part of the final sample; minor wording changes were made, and pilot interviews were not analyzed. The full interview guide is provided in Supplementary File S2. To promote consistency across sites, the two interviewers co-conducted the first two interviews, used the same guide and probing approach at every site, and met weekly during data collection to compare field notes and probing practices; the core questions were not changed after piloting, although probes were adapted to each participant’s role.

Interviews were conducted face-to-face in private rooms at participants’ workplaces (n = 24) or by secure video call (n = 6), according to participant preference, in English, Arabic, or a combination of both. Interviewers were R.M.A. (male; PhD, pharmaceutical sciences) and S.I.A. (male; MD, family and community medicine), both experienced in qualitative interviewing. R.M.A. conducted the interviews with clinical pharmacists and nurses (n = 16), and S.I.A. conducted those with physicians and governance leads (n = 14). Both interviewers are university faculty members; neither held a clinical, supervisory, managerial, or evaluative role in relation to any participant. Some participants knew the interviewers through academic or continuing-education activities; neither interviewer had a line-management or teaching relationship with any participant at the time of this study. Before consent, participants were informed of the interviewers’ professional backgrounds and of the study aims. M.S. did not conduct interviews and had no contact with participants or sites. Interviews were audio-recorded with permission, and field notes were written immediately afterward. Interviews lasted a mean of 52 min (range 38–74 min). Recordings were transcribed verbatim; Arabic-language interviews and Arabic-language portions of bilingual interviews were transcribed in Arabic and translated into English by a bilingual researcher, with a second bilingual researcher checking a random sample of translated passages against the original audio for conceptual equivalence. Discrepancies were resolved by discussion, and quotations from Arabic-language interviews reported in this article were checked against the original Arabic transcripts by both interviewers. Transcripts were not returned to participants, but preliminary findings were shared for member reflection (Section 2.6).

### 2.5. Data Analysis

Data were analyzed using reflexive thematic analysis following the six phases described by Braun and Clarke [67,68]: familiarization, coding, generating initial themes, reviewing themes, defining and naming themes, and writing up. Analysis was managed in QualCoder (open-source software). Two researchers (M.S. and R.M.A.) read all transcripts and coded inductively at semantic and latent levels. To enrich interpretation rather than to establish coding reliability, they independently coded the first six transcripts and then met to discuss how their disciplinary positions (nursing and pharmacy) shaped what they noticed [69]. Independent coding was not used to establish inter-coder reliability or to reach consensus coding; no agreement statistics were calculated, consistent with the assumption in reflexive thematic analysis that coding reflects the researcher’s interpretive engagement with the data rather than a single correct reading [68,69]. Codes were clustered into candidate themes, which were reviewed against coded extracts and the full dataset and refined through team discussion with S.I.A. For example, codes such as “IV continued after oral intake resumed”, “waiting for the round to switch”, and “pharmacist switch is only a recommendation” were first clustered into a candidate theme on route inertia; on review, they were combined with codes such as “injection equals real treatment” and “no immediate-release oral morphine on the shelf” because together they explained why parenteral use persisted beyond clinical need, forming Theme 2. An analytic trail linking illustrative coded excerpts to codes, subthemes, and themes for all five themes is provided in Supplementary Table S1. In phase five, CFIR 2.0 domains and constructs were used deductively as a lens to locate each theme within the implementation context [60]; themes were not forced into the framework, and inductive content that did not map to a construct was retained. Finally, after the themes had been finalized, participant-proposed solutions were mapped to discrete strategies from the Expert Recommendations for Implementing Change (ERIC) compilation [61]. This was an author-led interpretive step: proposals were extracted from the coded dataset into a matrix with the barrier to which they referred, matched to ERIC strategies independently by R.M.A. and M.S. using the published strategy definitions, discussed with S.I.A. until agreement was reached, and checked against the source excerpts. The mapping was not validated by participants or external implementation experts. Cross-site and cross-role comparisons were made throughout. A site-by-theme matrix summarized, for each site and professional group, how each candidate theme was expressed; this matrix was used to distinguish barriers described at all sites from site- or sector-specific variations (for example, EHR functionality in the private hospital), which are reported where relevant.

### 2.6. Rigor and Reflexivity

Trustworthiness was addressed using Lincoln and Guba’s criteria as operationalized for thematic analysis [70]. Credibility was supported through prolonged engagement with the data, investigator triangulation across three disciplines, and member reflections [69]. Eight participants (two from each professional group, covering all four sites) were purposively invited because they had given detailed accounts and together represented each site and group; all eight accepted. Each received a two-page summary of the preliminary themes and discussed it individually with R.M.A. or S.I.A. (in person or by video call; approximately 20–30 min). Feedback was recorded in written notes that were entered into the audit trail; these sessions were not audio-recorded or analyzed as additional data, and feedback was treated as a further perspective rather than as verification. Two concrete changes followed. Theme 2, provisionally titled “Patients ask for the needle”, was broadened because pharmacist and nurse participants emphasized that formulary and supply constraints and absent step-down triggers, not only patient demand, sustained parenteral use; subtheme 2.3 was separated, and the theme was renamed “The needle is the treatment” to reflect a belief shared by staff as well as patients. Theme 5, provisionally titled “Pharmacists without authority”, was expanded after nurse participants described their own unmandated decisions about route and dose in multi-option “as needed” orders, which led to subtheme 5.2 and the current title. Dependability and confirmability were supported by an audit trail of coding decisions, theme maps, and analytic memos. Transferability was supported by a detailed description of the setting and sample. The research team comprised a pharmaceutical scientist (R.M.A.) and a family and community medicine physician (S.I.A.), both faculty members at King Faisal University in Al-Ahsa, and a gerontological nurse with expertise in qualitative methodology (M.S.) based at Cairo University, who contributed as the team’s methodologist through an established inter-university research collaboration, worked only with de-identified transcripts, and had no relationship with any site or participant. M.S.’s position outside this study hospitals was used deliberately to question assumptions that the locally embedded interviewers might take for granted, such as the normalcy of parenteral analgesia in SCD care. The team recognized that the interviewers’ professional identities might elicit profession-specific narratives (for example, pharmacists emphasizing their advisory role when interviewed by a pharmaceutical scientist) or encourage participants to present practice favorably to a physician interviewer. Interviewers wrote reflexive memos after each interview; the analysis compared accounts elicited by each interviewer, and quality was judged against published criteria for reflexive thematic analysis [69].

### 2.7. Ethical Considerations

This study was conducted in accordance with the Declaration of Helsinki and was approved by the Research Ethics Committee of King Faisal University (approval no. KFU-REC-2025-SEP-ETHICS2491). The KFU approval covered the multicenter protocol, including all four participating hospitals. In addition, each hospital reviewed the approved protocol through its own research or administrative office and granted site permission before recruitment began; the military hospital issued its own research approval, and the remaining hospitals granted facilitation or administrative approval. No site required a separate full ethical review. All participants received written study information and provided written informed consent before the interview, including consent for audio recording and publication of anonymized quotations. Participation was voluntary and unrelated to employment evaluation, and managers were not informed who participated. Transcripts were de-identified, and participants are identified by a code indicating participant number, professional group, and site (e.g., P14, clinical pharmacist, H2). Recordings and transcripts were stored on password-protected institutional drives accessible only to the research team.

## 3. Results

### 3.1. Participant Characteristics

Thirty participants took part: 11 physicians, 8 clinical pharmacists, 8 registered nurses, and 3 governance leads (a pharmacy and therapeutics committee chair, a medication safety officer, and a nursing quality director). Participants were distributed across H1 (n = 9), H2 (n = 8), H3 (n = 7), and H4 (n = 6). Their mean age was 39.6 years (SD 8.2), 18 were men, 19 were Saudi nationals, and they had a mean of 13.4 years (SD 7.1) of professional experience. Characteristics are summarized in Table 1. Governance perspectives were represented by three participants only, and no hospital executive was interviewed; findings about organizational governance therefore draw mainly on the accounts of frontline clinicians and middle managers.

**Table 1:** Characteristics of participants (N = 30).

Characteristic	Category	n (%) or Mean (SD)
Professional group	Physicians	11 (36.7%)
	Clinical pharmacists	8 (26.7%)
	Registered nurses	8 (26.7%)
	Governance leads ᵃ	3 (10.0%)
Study site	H1	9 (30.0%)
	H2	8 (26.7%)
	H3	7 (23.3%)
	H4	6 (20.0%)
Gender	Men	18 (60.0%)
	Women	12 (40.0%)
Age, years	Mean (SD); range	39.6 (8.2); 27–58
Nationality	Saudi	19 (63.3%)
	Non-Saudi	11 (36.7%)
Professional experience, years	Mean (SD)	13.4 (7.1)
Years at current hospital	Mean (SD)	7.2 (4.9)
Highest academic degree reported ᵇ	Bachelor’s degree	11 (36.7%)
	Master’s degree	7 (23.3%)
	Doctoral degree	5 (16.7%)
Professional qualification reported ᵇ	Clinical board certification	7 (23.3%)
Physician specialty (n = 11)	Emergency medicine	3 (27.3%)
	Anesthesia and acute pain	2 (18.2%)
	General surgery	2 (18.2%)
	Hematology	2 (18.2%)
	Oncology and palliative care	1 (9.1%)
	Internal medicine	1 (9.1%)
Nursing unit (n = 8)	Emergency department	2 (25.0%)
	Surgical wards	2 (25.0%)
	Oncology and hematology	2 (25.0%)
	Critical care and post-anesthesia care	2 (25.0%)
Interview language	English	21 (70.0%)
	Arabic	6 (20.0%)
	English and Arabic	3 (10.0%)
Interview mode	Face to face	24 (80.0%)
	Secure video call	6 (20.0%)

ᵃ Governance leads comprised the pharmacy and therapeutics committee chair, medication safety officer, and nursing quality director. ᵇ Qualification entries represent distinct categories in the available participant summary (11 bachelor’s, 7 master’s, 5 doctoral, and 7 clinical board certifications; N = 30). Clinical board certification is a professional credential, not an academic degree; the academic-degree classifications of the seven board-certified participants were not recorded separately in the available summary. Percentages use N = 30 except physician specialty (n = 11) and nursing unit (n = 8). SD, standard deviation.

### 3.2. Overview of Themes

**Figure 1:**
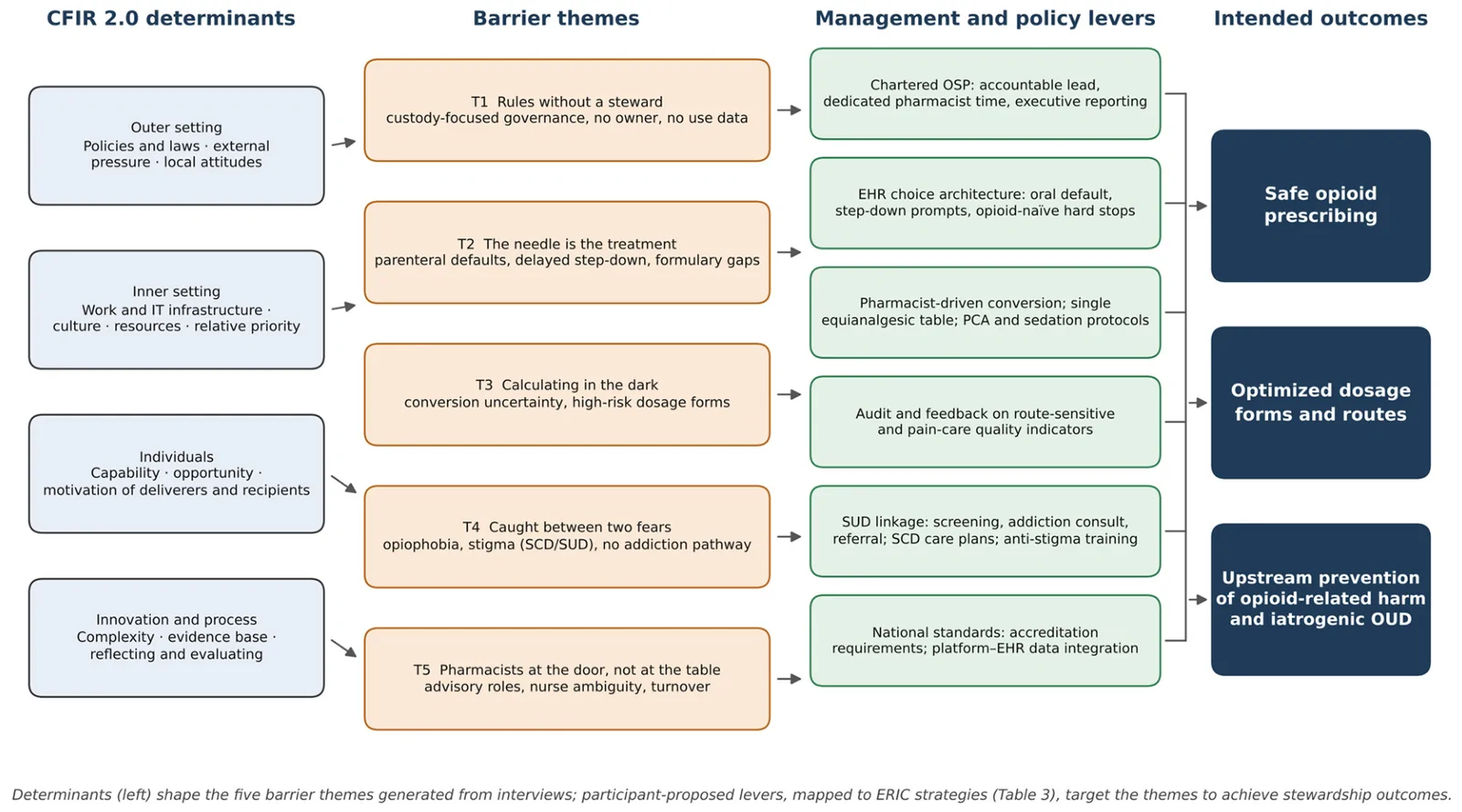
Conceptual model linking CFIR 2.0 determinants, the five barrier themes, participant-proposed management and policy levers, and hypothesized stewardship outcomes, which were not measured in this study. CFIR 2.0, updated Consolidated Framework for Implementation Research; EHR, electronic health record; OSP, opioid stewardship program; OUD, opioid use disorder; PCA, patient-controlled analgesia; SCD, sickle cell disease; SUD, substance use disorder.

Five themes were generated that describe the barriers to safe opioid prescribing and to dosage-form and route optimization (Table 2). Together, participants’ accounts depicted hospitals in which controlled-drug regulation was experienced as robust, but stewardship, understood as the active, data-driven optimization of opioid use, had no identifiable organizational owner. Participants at all four sites described these barriers, although their expression differed by sector; for example, participants from the private hospital (H3) described more advanced EHR functionality, whereas participants from the Ministry of Health sites described greater dependence on paper-based or hybrid workflows for controlled-drug documentation. Participant-proposed levers are presented in Section 3.8 and Table 3, and the relationships among determinants, themes, and levers are summarized in Figure 1. Unless otherwise stated, statements in the following sections about policies, formularies, EHR functions, safeguards, and practices are participant-reported and were not verified against documents or records. An analytic trail with illustrative excerpts for each subtheme is provided in Supplementary Table S1.

**Table 2:** Themes, subthemes, and corresponding CFIR 2.0 domains and constructs.

Theme	Subthemes	Core Barrier	CFIR 2.0 Domains (Constructs)
1. Rules without a steward	1.1 Regulation built for custody, not for optimization 1.2 Stewardship without a mandate 1.3 Data that count but do not inform	Controlled-drug governance focuses on custody and traceability; no accountable owner or use data for stewardship	Outer setting (policies and laws; external pressure); inner setting (work infrastructure; IT infrastructure; relative priority); process (reflecting and evaluating)
2. The needle is the treatment	2.1 A shared belief in the potency of injection 2.2 Step-down that waits for someone to remember 2.3 A formulary that shapes the route	Parenteral use persists beyond clinical need because of expectations, absent triggers, and supply constraints	Inner setting (culture: recipient-centeredness; materials and equipment; compatibility); individuals (innovation recipients: need, motivation; deliverers: opportunity); innovation (relative advantage)
3. Calculating in the dark	3.1 No shared equianalgesic language 3.2 High-risk forms without high-reliability systems 3.3 Transitions without a plan	Conversion, transdermal, extended-release, PCA, and discharge decisions rely on individual knowledge	Innovation (complexity; evidence base); individuals (capability); inner setting (access to knowledge and information; IT infrastructure)
4. Caught between two fears	4.1 Fear of addiction and scrutiny 4.2 Fear of under-treatment and complaints 4.3 Labels that follow patients 4.4 Stewardship that stops at the hospital door	Opiophobia and complaint pressure produce inconsistent practice; stigma toward patients with SCD or SUD; no OUD pathway	Outer setting (local attitudes; policies and laws; partnerships and connections); individuals (motivation; capability); inner setting (culture)
5. Pharmacists at the door, not at the table	5.1 Recommendations without authority 5.2 Nurses deciding without a mandate 5.3 Workforce turnover and training gaps	Advisory pharmacist roles, ambiguous nursing orders, and turnover limit stewardship capacity	Inner setting (work infrastructure; relational connections; communications; available resources); individuals (opportunity; capability)

CFIR 2.0, updated Consolidated Framework for Implementation Research [60]; IT, information technology; OUD, opioid use disorder; PCA, patient-controlled analgesia; SCD, sickle cell disease; SUD, substance use disorder.

### 3.3. Theme 1: “Rules Without a Steward”: Compliance-Oriented Governance Without Stewardship Ownership

#### 3.3.1. Subtheme 1.1: Regulation Built for Custody, Not for Optimization

Participants across all sites described controlled-drug governance as rigorous with respect to custody: storage, counting, registers, witnessed wastage, and dispensing documentation. This regulatory architecture was valued for preventing diversion, but participants consistently distinguished it from stewardship. Compliance activity consumed time and attention and was audited closely, whereas the appropriateness of the opioid, dose, dosage form, or route was rarely examined.

*“We are excellent at counting. If one ampoule is missing, the whole hospital knows by the evening. But if a patient stays on IV morphine for five days when he could have taken tablets on day two, nobody will ever ask about it.” (P15, clinical pharmacist, H1)*

*“The inspections ask: is the cupboard locked, is the register signed, are the two signatures there? They never ask: was this the right opioid for this patient?” (P24, registered nurse, H4)*

Participants described the national electronic platform for controlled-drug prescribing as a positive step toward traceability, but several reported a transitional period in which the national platform and hospital EHRs operated in parallel, requiring duplicate entry and limiting visibility of prescriptions issued in other facilities.

#### 3.3.2. Subtheme 1.2: Stewardship Without a Mandate

No participant could identify a person or committee accountable for opioid stewardship as a program. Opioid-related decisions were dispersed across pharmacy and therapeutics committees, medication safety committees, acute pain services, and palliative care teams, each acting within its own remit. Governance leads described the pharmacy and therapeutics committee as reactive, addressing formulary requests and incident reports rather than monitoring patterns of use.

*“Antimicrobial stewardship has a charter, a team, a dashboard, and it reports to the medical director every quarter. For opioids we have nothing like that. Opioids come to the committee only when there is an incident.” (P28, pharmacy and therapeutics committee chair, H2)*

*“Everyone owns a small piece. The pain team owns the epidurals, oncology owns their patients, the ER does its own thing. But who owns the whole picture? Honestly, nobody.” (P04, anesthesiologist, H3)*

#### 3.3.3. Subtheme 1.3: Data That Count but Do Not Inform

Participants reported that available data were designed to reconcile stock rather than to describe prescribing. According to participants, no site routinely measured indicators such as the proportion of parenteral opioid doses, time to intravenous-to-oral conversion, discharge morphine milligram equivalents, naloxone use, or opioid-related adverse events. Medication-error reporting was perceived as capturing only a small fraction of opioid-related harm.

*“I can tell you exactly how many vials of fentanyl we used last month. I cannot tell you how many patients were over-sedated.” (P29, medication safety officer, H1)*

### 3.4. Theme 2: “The Needle Is the Treatment”: Parenteral Defaults, Delayed Step-Down, and Formulary Gaps

#### 3.4.1. Subtheme 2.1: A Shared Belief in the Potency of Injection

Participants described a pervasive belief, held by patients, families, and many staff, that injected opioids are stronger, faster, and more legitimate than oral forms. This belief was most visible in the emergency department and in the care of adults with SCD, where patients’ expectations of intravenous analgesia were shaped by years of experience. Two nurses and one emergency physician reported that intramuscular injections were still requested and occasionally given, despite guidance discouraging this route.

*“The patient says, ‘Tablets do nothing for me, I need the needle.’ The family is standing there. If you give tablets, they think you are refusing to treat him.” (P02, emergency physician, H1)*

*“Some of our sickle cell patients have been coming since they were children. For them, the IV is the treatment. Changing that is not one conversation; it is years of trust.” (P09, hematologist, H4)*

#### 3.4.2. Subtheme 2.2: Step-Down That Waits for Someone to Remember

Once intravenous opioids were started, conversion to oral therapy depended on an individual physician noticing that the patient could tolerate oral intake and actively changing the order. Participants described no automatic triggers, no pharmacist authority to convert, and weekend and nighttime delays. Nurses described continuing “as needed” intravenous doses because no oral alternative had been prescribed.

*“The patient is eating a full lunch and I am still giving IV morphine PRN, because that is what is ordered. I page the doctor, the doctor says ‘tomorrow on the round’. Tomorrow becomes three days.” (P21, registered nurse, H2)*

*“For antibiotics we have an IV-to-oral policy and the pharmacist can switch. For opioids, if I recommend a switch, it is a recommendation. Maybe it is accepted, maybe not.” (P13, clinical pharmacist, H3)*

#### 3.4.3. Subtheme 2.3: A Formulary That Shapes the Route

Participants described formulary and supply constraints that pushed practice toward parenteral or less suitable products. Pharmacists at two sites reported the absence of immediate-release oral morphine or oxycodone in suitable strengths, the lack of oral liquid formulations for patients with swallowing difficulty, and intermittent shortages of oral products that led to substitution with injectable forms. Participants reported that extended-release products were sometimes the only oral strong-opioid option on the ward, which created pressure to use them in opioid-naïve patients.

*“If the only strong oral opioid on the shelf is the long-acting one, the doctor has two choices: keep the patient on IV, or give a long-acting tablet to someone who has never had opioids before. Both choices are wrong.” (P17, clinical pharmacist, H4)*

### 3.5. Theme 3: “Calculating in the Dark”: Conversion Uncertainty and High-Risk Dosage Forms

#### 3.5.1. Subtheme 3.1: No Shared Equianalgesic Language

Participants reported that each department, and sometimes each clinician, used a different equianalgesic table drawn from textbooks, mobile applications, or memory. Participants were not aware of an approved institutional conversion table at any site with explicit cross-tolerance reductions, and physicians outside anesthesia and palliative care expressed low confidence in conversions, particularly for fentanyl and methadone. Pharmacists described intercepting conversion errors, but only when they happened to review the order.

*“I asked three doctors to convert the same patch to oral morphine and got three different answers. None of them was dangerous that day, but one day it will be.” (P16, clinical pharmacist, H2)*

*“Honestly, when I convert IV to oral, I look it up on my phone. Which table is correct, I am not sure. Nobody taught us this properly.” (P07, general surgeon, H3)*

#### 3.5.2. Subtheme 3.2: High-Risk Forms Without High-Reliability Systems

Participants identified transdermal fentanyl, PCA, and extended-release products as dosage forms whose risks were not matched by protective systems. Pharmacists at three sites described fentanyl patches being initiated in opioid-naïve patients, often by teams unfamiliar with their delayed onset and prolonged action. PCA was described as dependent on individual nurses’ expertise, with variable double-checking of pump programming, inconsistent sedation scoring, and limited access to continuous capnography outside critical care.

*“The patch looks gentle. It is just a sticker. So it is given to an elderly patient who was never on opioids, and two days later we are calling the rapid response team.” (P18, clinical pharmacist, H1)*

*“For PCA, the safety is me. I check the concentration, I check the lockout, I watch the breathing. If I am busy with five other patients, the safety is less.” (P23, registered nurse, H1)*

#### 3.5.3. Subtheme 3.3: Transitions Without a Plan

Participants described discharge as a point at which route and dosage-form decisions were rarely reviewed. Discharge quantities were based on habit rather than on in-hospital consumption, tapering plans were uncommon, and patients discharged on extended-release or transdermal products often lacked clear instructions or follow-up.

*“We write ‘tramadol for two weeks’ because that is what we always write. Nobody looks at how much the patient used in the last 24 h.” (P06, general surgeon, H2)*

### 3.6. Theme 4: “Caught Between Two Fears”: Opiophobia, Stigma, and the Missing Addiction Pathway

#### 3.6.1. Subtheme 4.1: Fear of Addiction and Scrutiny

Many prescribers and nurses described fear of causing addiction, of diversion, and of regulatory or legal scrutiny. This fear produced avoidance (prescribing weak opioids or none), under-dosing, and an aversion to oral take-home supplies, which, paradoxically, sustained reliance on supervised parenteral doses within the hospital.

*“With the injection, I give it in front of me, it is documented, finished. With tablets for home, if something happens, it is my name on the prescription. So many doctors prefer to keep the patient inside on injections.” (P01, emergency physician, H2)*

#### 3.6.2. Subtheme 4.2: Fear of Under-Treatment and Complaints

Simultaneously, participants described pressure from patients, families, and patient-experience processes to provide rapid and potent analgesia. Complaints about pain were perceived to carry institutional consequences, and in the absence of shared protocols, clinicians oscillated between restriction and escalation.

*“On one side, the fear of addiction. On the other side, the fear of complaints. In between, there is no protocol to protect the doctor, so each one decides alone.” (P10, internal medicine physician, H4)*

#### 3.6.3. Subtheme 4.3: Labels That Follow Patients

Participants, particularly nurses and hematologists, described labeling frequent attenders, most often adults with SCD, as “drug-seeking”. These labels were perceived to delay analgesia, encourage restrictive or inconsistent dosing, and erode trust, which in turn reinforced patients’ insistence on parenteral routes. Participants reported that individualized pain plans for patients with frequent crises were available at only one site.

*“When a known sickle cell patient arrives, someone at the station says, ‘Your friend is back.’ From that moment, the patient is not a patient in pain; he is a problem.” (P26, registered nurse, H4)*

#### 3.6.4. Subtheme 4.4: Stewardship That Stops at the Hospital Door

Participants recognized that some patients showed signs of problematic opioid use, but they described no institutional pathway to assess or respond to OUD. According to participants, screening was not routine, addiction specialists were not available for inpatient consultation, and referral to specialized addiction services was perceived as slow and stigmatizing for patients. Participants noted that MOUD options were not available in their hospitals, so recognition of OUD often led to restriction rather than treatment.

*“If I suspect addiction, what can I do? Reduce the dose and write a note. There is no consult, no pathway, no medication we can start. So we restrict, and the patient goes to another hospital.” (P11, hematologist, H1)*

### 3.7. Theme 5: “Pharmacists at the Door, Not at the Table”: Professional Boundaries and Workforce Constraints

#### 3.7.1. Subtheme 5.1: Recommendations Without Authority

Clinical pharmacists described their role in opioid stewardship as advisory and largely invisible. Recommendations on route, dose, or dosage form were often accepted, but they depended on relationships with individual physicians and on pharmacists’ presence on ward rounds, which was limited by staffing.

*“I can see the problem from the pharmacy screen, but I am not at the bedside and not on the round. By the time my recommendation reaches the team, the decision is already made.” (P19, clinical pharmacist, H3)*

#### 3.7.2. Subtheme 5.2: Nurses Deciding Without a Mandate

Nurses described themselves as de facto decision-makers about which “as needed” opioid, route, and dose a patient received, because orders frequently listed multiple options. They felt responsible for these choices without protocols, sedation-scale standards, or the authority to request conversion.

*“The order says morphine IV or oral, two to five milligrams, as needed. So I am choosing the route and the dose, but I have no protocol that tells me how.” (P22, registered nurse, H3)*

#### 3.7.3. Subtheme 5.3: Workforce Turnover and Training Gaps

Across sites, participants described frequent staff rotation and turnover, especially among internationally recruited staff, limited acute pain service coverage at night and at weekends, and the absence of structured interprofessional training on opioid conversion, monitoring, or stigma. Knowledge therefore resided in individuals rather than in systems.

*“Every year we train a new group of nurses on PCA, and every year half of them leave. The knowledge walks out of the door with them.” (P30, nursing quality director, H2)*

### 3.8. Participant-Proposed Management and Policy Levers

Participants proposed solutions at the hospital, unit, inter-organizational, and national policy levels (Table 3). The proposals raised most consistently across sites and professional groups were a formally chartered OSP with an accountable lead, dedicated pharmacist time, and regular reporting to executive leadership, modeled on existing antimicrobial stewardship structures; EHR redesign that makes the oral route the default when appropriate, embeds maximum doses and opioid-naïve safeguards for transdermal and extended-release products, and prompts review of parenteral orders after a defined interval; a pharmacist-supported intravenous-to-oral conversion protocol supported by a single institutional equianalgesic table; standardized sedation assessment and PCA double-checking, with targeted capnography; audit and feedback on a small set of stewardship indicators; individualized pain plans for patients with frequent SCD crises; and an SUD pathway linking screening, inpatient consultation, and referral to specialized addiction services. At the policy level, participants called for national OSP standards to be incorporated into accreditation requirements and for integration of the national controlled-drug prescribing platform with hospital EHRs. These proposals are reported as participants’ suggestions; their mapping to implementation strategies in Table 3 is the authors’ interpretation (Section 2.5), and none was implemented or evaluated in this study.

*“We do not need more rules about locks. We need someone whose job is to look at how opioids are actually used, with data, every month, and the power to change the order sets.” (P28, pharmacy and therapeutics committee chair, H2)*

**Table 3:** Participant-proposed management and policy levers, mapped by the authors to barriers, ERIC implementation strategies, and level of action.

Barrier (Theme)	Proposed Lever	ERIC Strategies	Level of Action ᵃ	Literature (Type) ᵇ
No accountable owner (1.2)	Charter an OSP within pharmacy and therapeutics governance, with a physician–pharmacist lead, dedicated pharmacist time, and quarterly reporting to executive leadership, adapting the antimicrobial stewardship template	Recruit, designate, and train for leadership; create new clinical teams; develop a formal implementation blueprint	Hospital	[13,14,18,47] C
Data reconcile stock, not use (1.3)	Core indicator set (parenteral share of opioid doses, time to intravenous-to-oral conversion, discharge MME, naloxone administrations, opioid-related adverse events, pain-care quality) with unit-level feedback	Develop and implement tools for quality monitoring; audit and provide feedback	Hospital	[71] E; [72] C
Parenteral defaults and delayed step-down (2.1–2.2)	Order sets defaulting to the oral route when enteral intake is documented and no exclusion applies; review prompt for parenteral opioids after a defined interval; pharmacist-supported intravenous-to-oral conversion protocol (safeguards in Section 4)	Change record systems; remind clinicians; revise professional roles	Hospital	[10,11,21] E; [22] C
Formulary gaps (2.3)	Formulary review to ensure immediate-release oral options, oral liquids, and suitable strengths; shortage guidance favoring oral equivalents	Conduct local consensus discussions; change physical structure and equipment	Hospital	[8] G; [33] C
Conversion uncertainty (3.1)	Single institutional equianalgesic table with cross-tolerance reduction embedded in the EHR; pharmacist verification for rotations involving fentanyl or methadone	Develop educational materials; change record systems	Hospital	[23,24] C; [25] G
High-risk dosage forms (3.2)	Hard stops, with documented specialist override, for transdermal fentanyl and extended-release opioids in opioid-naïve patients; standardized PCA protocols with independent double-check; sedation scale for all patients receiving opioids; risk-based capnography	Change record systems; change physical structure and equipment; develop and implement tools for quality monitoring	Hospital	[2,28,29] C; [16] G
Transitions without a plan (3.3)	Discharge quantity guide based on in-hospital use; tapering instructions; naloxone co-prescribing for high-risk patients	Remind clinicians; intervene with patients/consumers to enhance uptake and adherence	Hospital	[4] C; [12,17] E
Opiophobia and complaint pressure (4.1–4.2)	Interprofessional education on pain assessment, multimodal opioid-sparing analgesia, and opioid safety; protocols that protect clinicians acting within guidance	Conduct ongoing training; develop educational materials	Hospital (unit/team)	[8,73] G; [37] C
Stigma toward patients with SCD (4.3)	Individualized care plans for frequent vaso-occlusive crises; time-to-first-analgesia targets; anti-stigma training	Conduct educational meetings; involve patients/consumers and family members; develop and implement tools for quality monitoring	Hospital (unit/team)	[54,55,57] C
No addiction pathway (4.4)	OUD screening, inpatient addiction consultation, and referral to specialized addiction services; national policy dialogue on MOUD	Create new clinical teams; develop resource-sharing agreements; mandate change	Hospital; inter-organizational; national policy	[50,51,74] C; [75] E
Advisory pharmacists and ambiguous nursing orders (5.1–5.2)	Collaborative practice agreements, within national scope-of-practice regulation, authorizing pharmacist-initiated route conversion and dose adjustment under a physician-approved protocol; nurse-driven algorithms for as-needed opioids with defined route and dose criteria	Revise professional roles; create new clinical teams	Hospital; national policy	[15,19,20] C
Turnover and training gaps (5.3)	Competency-based onboarding for PCA, conversion, and sedation monitoring; knowledge embedded in order sets rather than individuals	Conduct ongoing training; make training dynamic	Hospital	[16] G; [38] C
Fragmented data; no external requirement (1.1, 1.3)	Integrate the national controlled-drug prescribing platform with hospital EHRs; include OSP structures and route indicators in national accreditation standards	Facilitate relay of clinical data to providers; change accreditation or membership requirements	Inter-organizational; national policy	[9] E; [46] C

ERIC, Expert Recommendations for Implementing Change [61]; EHR, electronic health record; MME, morphine milligram equivalents; MOUD, medication for opioid use disorder; OSP, opioid stewardship program; OUD, opioid use disorder; PCA, patient-controlled analgesia; SCD, sickle cell disease. ᵃ Hospital, actions within a single organization, including unit- or team-level actions; inter-organizational, actions requiring agreements between hospitals, health clusters, or specialized services; national policy, actions requiring regulatory, accreditation, or ministry-level decisions. ᵇ E, empirical evidence on the effect of the same or a closely analogous intervention; G, guideline, consensus statement, or expert recommendation; C, contextual or descriptive evidence (surveys, qualitative, epidemiological, or regulatory sources) that indicates relevance but not effectiveness. Mapping procedure: participant-proposed levers were extracted from the coded dataset, matched to ERIC strategies [61] independently by two authors (R.M.A. and M.S.) using the published definitions, discussed with the third author (S.I.A.) until agreement was reached, and checked against the source excerpts, informed by published guidance on matching barriers to implementation strategies [76]. The mapping is an author-led analytic interpretation; it was not validated by participants or external experts, and no lever was implemented or evaluated in this study.

## 4. Discussion

To our knowledge, this is one of the first qualitative studies to examine stakeholders’ perceptions of barriers to implementing opioid stewardship in Saudi hospitals, and one of few internationally to treat the optimization of opioid dosage forms and routes as an explicit stewardship target. Because it was conducted in four hospitals in a single region, its findings offer an in-depth account of this context rather than a representative picture of Saudi healthcare organizations. The central interpretation is a structural asymmetry, as perceived by participants: the four hospitals operated within a mature regulatory architecture for the custody and traceability of controlled drugs, yet, according to participants, lacked an accountable organizational structure for the clinical optimization of opioid use. The five themes describe how this asymmetry manifests across CFIR 2.0 domains, from outer-setting regulation and inner-setting governance to individual fears and professional boundaries, and the participant-proposed levers suggest how it might be addressed.

These findings differ from, and add to, previous work in three ways. First, earlier Saudi studies have largely examined individual clinicians’ knowledge and attitudes or described prescribing patterns [35,36,37,38,39,40,41]; by focusing on organizational structures, the present study locates the problem less in individual knowledge deficits than in the perceived absence of accountable ownership, data, and protocols. Second, international implementation studies of OSPs have mostly been conducted in North American or Australian hospitals in which programs already existed [14,18,19,20]; this study describes a pre-implementation context in which controlled-drug regulation is strong, but no chartered program exists, which may resemble that of other health systems now considering stewardship. Third, by treating dosage forms and routes as stewardship decisions, this study identifies route-specific mechanisms, including shared beliefs about the potency of injection, step-down that depends on individual memory, formulary-driven route choices, and high-risk products without system safeguards, that have received little attention in a stewardship literature focused mainly on prescribing quantity and duration [6,10,11,12].

The first theme echoes international evidence that OSPs vary widely in structure and that leadership endorsement, defined roles, and resourcing determine whether they are sustained [14,18]. What was distinctive in participants’ accounts was the contrast with antimicrobial stewardship, which participants described as chartered, staffed, and reported to leadership. Regional survey data show that antimicrobial stewardship is now common in Gulf hospitals, even though pharmacists’ clinical roles within these programs remain limited [47]. This existing infrastructure, together with the framing of opioid stewardship as an extension of antimicrobial stewardship principles [13], suggests a potentially available management lever: OSPs could be built on existing governance templates rather than created de novo, although the feasibility and effectiveness of this transfer require evaluation. The finding that available data reconcile stock rather than describe use is similarly actionable. Audit and feedback produce modest but consistent improvements in professional practice, particularly when baseline performance is low and feedback is repeated and paired with explicit targets [71]; a small set of route-sensitive indicators could make stewardship performance visible in these hospitals.

The second and third themes extend the stewardship literature by showing how dosage-form and route decisions are shaped by organizational rather than purely clinical factors. A shared belief in the superior potency of injection, delayed step-down that depends on individual memory, and formulary gaps together sustained parenteral use beyond clinical need. Evidence that oral opioids can achieve analgesia comparable to intravenous PCA when patients tolerate oral intake [21], together with experience from antimicrobial intravenous-to-oral switch programs, which are limited mainly by clinicians’ beliefs about bioavailability [22], suggests that protocolized, pharmacist-supported step-down might be adapted for opioids, provided that appropriate safeguards are developed and locally evaluated. The persistence of intramuscular administration reported by some participants mirrors earlier findings from an Al-Ahsa emergency department [54] and indicates that route optimization in SCD care remains an unresolved quality issue. Participants’ accounts of inconsistent equianalgesic conversion are consistent with evidence that clinicians’ conversions of identical regimens vary widely and that conversion tables have limited empirical support [23,24,26]; expert recommendations to apply cross-tolerance reductions and individualized titration [25,27] could be operationalized in a single institutional table embedded in the EHR. Reports of transdermal fentanyl initiation in opioid-naïve patients align with population data showing that most fentanyl patch initiations occur without adequate prior opioid exposure [28], and descriptions of PCA safety as dependent on individual nurses reflect the error patterns documented in national reporting systems [29] and the preventable respiratory events identified in closed-claims analyses [2]. Read alongside this literature, participants’ accounts point to system-level safeguards, such as EHR hard stops, standardized pump protocols, and risk-based monitoring [16], rather than reliance on individual vigilance, as candidates for implementation; their effectiveness in Saudi hospitals has not been established.

These route and dosage-form findings have direct implications for patient safety. Opioids are high-alert medications. Respiratory depression, usually preceded by progressive sedation, is their most serious acute adverse effect and is more likely in opioid-naïve and older patients, with concomitant sedatives, after dose escalation, and during transitions between agents or routes [2,77]. Conversion errors, PCA programming errors, and initiation of transdermal fentanyl in opioid-naïve patients, all described by participants, are recognized mechanisms of overdose and harm [23,28,29], and opioid-related adverse events in hospitalized patients are associated with longer stays and higher costs [78]. Under-treatment carries its own risks: inadequately managed acute pain is associated with patient suffering, delayed mobilization, cardiopulmonary and thromboembolic complications, and the development of persistent pain [79], and delayed analgesia in vaso-occlusive crises compounds distress and erodes trust [54,57]. Participants’ accounts of opiophobia alongside pressure for rapid parenteral relief suggest that both types of risk may coexist in these hospitals. Because this study did not measure medication errors, adverse events, or pain outcomes, these links remain hypotheses that stewardship evaluations should test with objective data.

Route optimization should therefore not be equated with avoiding parenteral opioids. Parenteral, usually intravenous, administration remains appropriate when patients cannot take or absorb oral medicines (for example, nil-by-mouth status, persistent vomiting, ileus, or reduced consciousness), when severe acute pain requires rapid titration, as in many vaso-occlusive crises, or when oral therapy has not provided adequate relief [8,80]. For people with SCD, international guidelines recommend assessment and administration of analgesia within one hour of emergency department arrival, reassessment every 30–60 min, and opioid dosing tailored to the patient’s baseline opioid use and previously effective therapy [80]; any default or step-down rule must therefore preserve timely relief and allow clinicians to follow individualized care plans. Conversely, intravenous-to-oral conversion is more appropriate when enteral intake and absorption are established, pain is controlled or improving on a stable parenteral requirement, and a suitable immediate-release oral product is available [8]. Conversion should be based on the patient’s actual parenteral requirement over the preceding 24 h, apply a cross-tolerance reduction when changing agents, include rescue doses, and be followed by reassessment of pain, sedation, and respiration [16,25,27]. Hard-stop alerts can prevent serious errors but are also associated with alert fatigue, workarounds, and delays in treatment [81]; a hard stop for transdermal fentanyl or extended-release opioids in opioid-naïve patients should therefore allow a documented, specialist-authorized override, for example, by palliative care or acute pain services. Table 4 presents an author-developed, non-validated, practice-oriented framework that connects participant-proposed strategies with clinical safeguards and professional roles synthesized from published guidance and the authors’ interpretation. It is not a direct representation of the interview data, and it was neither implemented nor tested in the participating hospitals.

**Table 4:** Author-developed, non-validated, practice-oriented framework for clinical safeguards and professional roles related to proposed opioid route and dosage-form strategies.

Participant-Proposed Strategy (Theme)	Clinical Safeguards and Exclusions	Physician Role	Pharmacist Role	Nurse Role
Oral-route default in opioid order sets (Theme 2)	Applies only when enteral intake and absorption are documented; parenteral route retained for nil-by-mouth status, persistent vomiting, ileus, reduced consciousness, or severe pain requiring rapid titration; one-step opt-out with documented reason; must not delay the first analgesic dose [8,80]	Selects the route for each patient; documents the clinical reason for parenteral use	Maintains order-set content; reviews opt-out reasons during audit	Assesses ability to swallow and tolerate oral intake; reports readiness for oral therapy
Pharmacist-supported intravenous-to-oral conversion (Themes 2, 3, 5)	Within a physician-approved protocol or collaborative practice agreement; based on the 24-h parenteral requirement using one institutional table; cross-tolerance reduction when changing agents; oral rescue dose; reassessment of pain, sedation, and respiration after the switch; prescriber notified and able to reverse [8,16,25,27]	Approves the protocol; retains prescribing responsibility; reviews and can reverse conversions	Identifies eligible patients; performs or verifies the equianalgesic calculation; recommends or enacts the switch within the protocol	Reassesses pain and sedation after the switch; escalates inadequate relief or excess sedation
Hard stops for transdermal fentanyl and extended-release opioids in opioid-naïve patients (Theme 3)	Opioid tolerance defined according to product labeling; documented override for specialist-supervised use (e.g., palliative care or acute pain service); monitoring of alert burden, override rates, and treatment delays [28,81]	Documents prior opioid exposure; seeks specialist authorization for override	Verifies opioid tolerance before dispensing; audits overrides	Confirms the monitoring plan; observes for delayed sedation after patch application
Standardized patient-controlled analgesia (Theme 3)	Standard drug concentrations and pump libraries; independent double-check of programming; standardized sedation and respiratory monitoring; capnography for patients at high risk [16,29]	Prescribes from a standard PCA order set	Prepares standard concentrations; reviews PCA orders	Performs the independent double-check; monitors sedation and respiration; escalates concerns
Individualized pain plans for people with sickle cell disease (Theme 4)	Plan developed with the patient and hematology team, specifying agent, dose, and route; analgesia within 1 h of arrival and reassessment every 30–60 min; route changes agreed with the patient rather than imposed by defaults [80]	Develops and updates the plan with the patient; authorizes deviations	Supports dose individualization and conversion when the route changes	Applies the plan at triage and on the ward; reassesses pain; challenges stigmatizing language
Discharge prescribing and tapering (Theme 3)	Quantity based on in-hospital use in the preceding 24 h; written tapering plan; naloxone and education for patients at high risk [12,17]	Prescribes quantity and taper; arranges follow-up	Reconciles discharge opioids; counsels the patient; supplies naloxone where indicated	Provides education on dosing, adverse effects, and safe storage

Note: This table is an author-developed, non-validated, practice-oriented framework. The proposed strategies are linked to barriers and suggestions identified in the interviews, whereas the detailed clinical safeguards, exclusions, and professional-role assignments were synthesized by the authors from external guidance and clinical interpretation. These details are not direct interview findings. The framework was not implemented, formally validated, or evaluated in this study and is not a clinical protocol; local multidisciplinary review, adaptation, and prospective evaluation would be required before use. h, hour; PCA, patient-controlled analgesia.

The fourth theme situates stewardship within the broader SUD prevention and care agenda. Participants described being caught between opiophobia and pressure to relieve pain, a tension also visible in Saudi data showing high national opioid consumption [34] alongside persistent knowledge deficits and fear of addiction among clinicians and trainees [35,36,37,38]. Without shared protocols, this tension was resolved individually, producing the prescribing variability reported in Saudi emergency and outpatient settings [39,40]. The labeling of patients with SCD as drug-seeking mirrors international evidence on emergency providers’ attitudes and longer waits for these patients [57,58]. In a region with the highest SCD prevalence in the Kingdom [52,53], and where inappropriate dosing in vaso-occlusive crisis is common [55], stigma may be not only an ethical concern but also a stewardship failure, because it may drive patients toward the parenteral routes that stewardship seeks to rationalize. Individualized pain plans and anti-stigma education could therefore be regarded as stewardship interventions. Equally important was participants’ description of stewardship that stops at the hospital door. Hospital-based addiction consultation services and emergency department-initiated buprenorphine improve engagement in treatment [74,75], and naloxone co-prescribing was associated with fewer opioid-related emergency visits in a nonrandomized study [17]. In Saudi Arabia, however, OUD treatment has historically been abstinence-based [50], and access to MOUD across the Arab region is constrained [51]. The absence of an inpatient pathway means that recognition of problematic use leads to restriction rather than treatment, potentially displacing patients to other facilities or to non-medical supply. Integrating screening, consultation, and referral into OSPs, and engaging in national policy dialogue on agonist therapy, could help align hospital stewardship with SUD prevention and care.

The role of patients deserves explicit attention. Participants frequently attributed parenteral use to the expectations of patients and families, but these attributions were professionals’ interpretations and may partly reflect stigma or defensive explanation rather than patients’ actual preferences. Without patients’ accounts, this study cannot determine whether requests for injection reflected inadequate relief from oral regimens, past experiences of delayed or under-dosed analgesia, concerns about adverse effects, or learned expectations. Changing default routes without involving patients risks being experienced as restriction and further eroding trust, particularly among adults with SCD. Shared decision-making about the route, individualized care plans developed with patients [80], and education about the onset and effectiveness of oral options are therefore integral to route optimization, and the perspectives of patients and families should be examined directly in future research.

The fifth theme highlights professional boundaries as a determinant of stewardship capacity. Pharmacists’ advisory, relationship-dependent influence and their workload constraints mirror findings from North American qualitative work [19] and contrast with programs in which pharmacist-led stewardship is formalized and valued by prescribers [15,20]. Nurses’ accounts of choosing among route and dose options without protocols extend Saudi evidence on nurses’ pain-management knowledge [37,38] by locating the problem in order design rather than solely in individual knowledge. Organizational barriers such as workload and weak interprofessional collaboration, previously reported among Saudi oncology nurses [42], and the workforce turnover described by participants suggest that durable improvement may require knowledge to be embedded in systems, such as order sets, protocols, and defaults, rather than in individuals.

### 4.1. Implications for Healthcare Management and Policy

Table 3 links each participant-proposed lever to discrete implementation strategies [61]. Because these levers were proposed by participants and mapped by the authors, and none were implemented or evaluated in this study, they are presented as candidate strategies for local adaptation and prospective evaluation rather than as proven solutions. They are grouped below by the level at which action would be required.

#### 4.1.1. Hospital-Level Actions

Within individual hospitals, participants’ accounts point to three candidate priorities: formal governance (a chartered OSP with an accountable lead and dedicated pharmacist time), choice architecture in the EHR, and measurement. EHR defaults have been associated with substantial changes in opioid prescribing without increases in refill requests [10,11], and evidence-based discharge prescribing guides can reduce excess supply [12]. Applying similar principles to route, by making oral administration the default when clinically appropriate and prompting review of parenteral orders, could be a low-cost extension, subject to local review and validation of the candidate safeguards outlined in Table 4. Implementation would require clear role allocation. Physicians would retain prescribing responsibility, approve conversion protocols, and document individualized exceptions; clinical pharmacists would identify patients eligible for step-down, perform or verify equianalgesic calculations, and audit prescribing against stewardship indicators; and nurses would assess readiness for oral therapy, apply standardized pain and sedation assessment, and escalate when relief is inadequate after a route change. An interprofessional OSP committee reporting to the medical director and linked to the pharmacy and therapeutics and medication safety committees would provide the governance within which these roles operate. Stewardship indicators should include the timeliness and quality of pain care as well as safety measures, so that stewardship is assessed as optimization rather than restriction [72].

#### 4.1.2. Inter-Organizational Actions

Several proposals extend beyond a single hospital. Referral from hospital-identified OUD to specialized addiction services, with agreed criteria, communication, and follow-up, would require formal agreements between hospitals and addiction treatment providers within the health cluster [74,75]. Sharing stewardship templates, conversion tables, and indicator definitions across the governmental, military, and private sectors in the region could reduce duplication, and a regional stewardship network could allow benchmarking. Integration of the national controlled-drug prescribing platform with hospital EHRs would give prescribers visibility of prescriptions across facilities, consistent with evidence that monitoring programs are associated with sustained reductions in high-risk prescribing [9], but would require technical interoperability and data-governance agreements between the platform operator and each hospital.

#### 4.1.3. National Policy Actions

Some participant proposals would require national decisions, and their feasibility in the Saudi regulatory and health-system context warrants critical examination. First, incorporating stewardship structures and route-optimization indicators into national accreditation standards [46] could accelerate adoption across sectors, but new standards would need to be developed and piloted by the accrediting body and would add to a compliance workload that participants already perceived as heavy. Second, expanded pharmacist authority for route conversion would depend on the scope of practice permitted by national regulation; because pharmacists’ prescribing roles in Gulf hospitals remain limited [47], collaborative, protocol-based conversion under physician oversight is a more feasible near-term model than independent pharmacist prescribing. Third, national protocols for OUD, including policy deliberation on medication for OUD, would need to be considered within the national legal framework for controlled substances and addiction treatment [43,50,51], which has historically emphasized abstinence-based care. These actions extend beyond what the present qualitative data can establish and are offered as directions for policy dialogue. Any reform must also preserve access to analgesia; given global inequities in opioid availability [32,33], the aim is optimization, not restriction.

### 4.2. Strengths and Limitations

Strengths include the multicenter design spanning governmental, military, and private sectors; the inclusion of prescribers, pharmacists, nurses, and governance leads; the use of an established implementation framework to connect findings to strategies; and an interdisciplinary research team. Several limitations should be considered. First, this study was conducted in four hospitals in one region with a distinctive SCD burden, and governance perspectives were represented by only three participants; no hospital executives or ministry-level policymakers were interviewed. The findings are therefore not representative of Saudi healthcare organizations as a whole. They may be transferable to tertiary hospitals with similar regulatory structures and patient populations, and readers can judge transferability using the contextual description provided, but they may apply less to primary care, to regions with lower SCD prevalence, or to hospitals with established stewardship programs. Second, the findings are based solely on interviews. Statements about the absence of stewardship programs, institutional conversion tables, indicators, formulary items, PCA safeguards, and individualized care plans, and about practices such as fentanyl patch initiation, reflect participants’ reports and were not verified against policies, formularies, EHR configurations, prescribing or dispensing records, incident reports, or direct observation. No data triangulation was undertaken, and this study did not measure prescribing patterns, medication errors, or patient-safety outcomes. Third, social desirability bias may have led participants to present their own practice favorably or to emphasize institutional shortcomings that reflected well on their professional group [82]; confidentiality assurances, recruitment that did not involve managers, and requests for concrete examples aimed to reduce, but cannot exclude, this bias. Participants who volunteered may also have held stronger views on stewardship than their colleagues. Fourth, the researchers’ positionality may have shaped both data generation and interpretation. Both interviewers are academic clinicians from professions represented in the sample, which may have encouraged profession-specific narratives or deference toward the physician interviewer [83]; reflexive memos, the involvement of an analyst external to the study sites, and comparison of accounts across interviewers were used to examine these influences. Fifth, nine interviews were conducted wholly or partly in Arabic; despite translation by bilingual researchers and checking against the original audio, culturally specific meanings may have been attenuated [84]. Sixth, patients and families were not interviewed. Their preferences regarding route, their experiences of stigma, and their views on treatment effectiveness and adverse effects were represented only through professionals’ accounts, which may misattribute motives to patients; future research should include patients, particularly adults with SCD, and family members alongside professionals. Seventh, member reflection involved eight participants, and feedback was documented in notes rather than recorded, so it offers a limited additional perspective [85]. Finally, the participant-proposed levers and their mapping to implementation strategies were not tested. Table 4 is an author-developed, non-validated, practice-oriented framework informed by external guidance and author interpretation; its safeguards and role assignments should not be construed as direct interview findings or a tested clinical protocol. The feasibility, effectiveness, and unintended consequences of these candidate approaches in the Saudi context require prospective implementation and evaluation.

## 5. Conclusions

In four tertiary hospitals in the Al-Ahsa region, participants described a strong regulatory architecture for the custody and traceability of controlled drugs but reported lacking the organizational ownership, data, protocols, and professional roles required for opioid stewardship. The perceived barriers to safe prescribing and to the optimization of dosage forms and routes included a parenteral-default culture, unstandardized conversion practices, high-risk dosage forms without system safeguards, competing fears and stigma, absent addiction-care pathways, and constrained pharmacist and nursing roles. These findings describe participants’ perceptions and reported practices; they do not establish actual prescribing patterns, medication-error rates, or patient-safety outcomes, which should be examined using prescribing records, incident data, and direct observation. Participants identified potentially actionable strategies: chartering accountable OSPs built on existing antimicrobial stewardship templates; EHR defaults that favor oral therapy when clinically appropriate, with safeguards that preserve individualized and timely analgesia, particularly for people with SCD; pharmacist-supported conversion within physician-approved protocols; audit and feedback on route-sensitive indicators; and linkage to addiction services. These strategies, and the national policy changes that some participants proposed, warrant prospective implementation and evaluation before conclusions can be drawn about their effects on opioid safety or on the prevention of opioid use disorder.

## Data Availability

The interview transcripts generated and analyzed during this study are not publicly available because they contain information that could compromise participant confidentiality. De-identified excerpts are available from the corresponding author upon reasonable request and subject to approval by the King Faisal University Research Ethics Committee.

## Supplementary Materials

The following supporting information can be downloaded at https://www.mdpi.com/article/10.3390/healthcare14213806/s1, File S1: COREQ reporting checklist; File S2: Interview guide; Table S1: Analytic trail linking illustrative coded excerpts to codes, subthemes, and themes.

## Abbreviations

The following abbreviations are used in this manuscript: CFIR, Consolidated Framework for Implementation Research; COREQ, Consolidated Criteria for Reporting Qualitative Research; EHR, electronic health record; ERIC, Expert Recommendations for Implementing Change; IV, intravenous; MME, morphine milligram equivalents; MOUD, medication for opioid use disorder; OSP, opioid stewardship program; OUD, opioid use disorder; PCA, patient-controlled analgesia; SCD, sickle cell disease; SRQR, Standards for Reporting Qualitative Research; SUD, substance use disorder.
