# Supplementary material for: Barriers to Safe Opioid Prescribing in Al-Ahsa Hospitals: A Multicenter Qualitative Study
Source: Healthcare (Basel). 2026 Nov 7;14(21):3806. doi: 10.3390/healthcare14213806 (PMC13763524; doi:10.3390/healthcare14213806)
Supplement: Supplementary file 1 [file healthcare-14-03806-s001.zip › healthcare-4658367-supplementary.pdf]

Supplementary Materials

Barriers to Safe Opioid Prescribing in Al-Ahsa Hospitals: A Multicenter Qualitative Study

File S1. COREQ (Consolidated Criteria for Reporting Qualitative Research) checklist

Locations refer to section numbers of the revised manuscript, so they remain valid after typesetting.

No.	Item	Guide question/description	Reported in
Domain 1: Research team and reflexivity			
1	Interviewer/facilitator	Which author(s) conducted the interview or focus group?	2.4 (R.M.A. and S.I.A.)
2	Credentials	What were the researcher's credentials?	2.4 (PhD; MD); 2.6
3	Occupation	What was their occupation at the time of the study?	2.4; 2.6 (university faculty members)
4	Gender	Was the researcher male or female?	2.4 (both male)
5	Experience and training	What experience or training did the researcher have?	2.4; 2.6
6	Relationship established	Was a relationship established prior to study commencement?	2.4 (no supervisory or evaluative relationship; prior academic acquaintance reported)
7	Participant knowledge of the interviewer	What did the participants know about the researcher?	2.4 (professional background and study aims disclosed before consent)
8	Interviewer characteristics	What characteristics were reported about the interviewer/facilitator?	2.6; 4.2 (positionality, reflexive memos)
Domain 2: Study design			
9	Methodological orientation and theory	What methodological orientation was stated to underpin the study?	2.1 (qualitative description; CFIR 2.0 as sensitizing framework); 2.5 (reflexive thematic analysis)
10	Sampling	How were participants selected?	2.3 (purposive, maximum-variation)
11	Method of approach	How were participants approached?	2.3 (institutional e-mail via site coordinators; governance leads invited directly)
12	Sample size	How many participants were in the study?	2.3; 3.1 (N = 30)
13	Non-participation	How many people refused to participate or dropped out? Reasons?	2.3 (7 of 37 volunteers did not proceed; reasons given; non-responders not quantifiable)
14	Setting of data collection	Where was the data collected?	2.4 (private rooms at workplace or secure video call)
15	Presence of non-participants	Was anyone else present besides the participants and researchers?	2.4 (private rooms; no others present)
16	Description of sample	What are the important characteristics of the sample?	3.1; Table 1

No.	Item	Guide question/description	Reported in
17	Interview guide	Were questions, prompts, guides provided by the authors? Was it pilot tested?	2.4; File S2 (piloted with two clinicians)
18	Repeat interviews	Were repeat interviews carried out?	No; single interviews (2.4)
19	Audio/visual recording	Did the research use audio or visual recording?	2.4 (audio-recorded)
20	Field notes	Were field notes made during and/or after the interview?	2.4 (immediately after)
21	Duration	What was the duration of the interviews?	2.4 (mean 52 min; range 38–74)
22	Data saturation	Was data saturation discussed?	2.3 (information power and qualitative adequacy criteria used instead of saturation)
23	Transcripts returned	Were transcripts returned to participants for comment and/or correction?	2.4 (not returned; member reflection on preliminary themes, 2.6)
Domain 3: Analysis and findings			
24	Number of data coders	How many data coders coded the data?	2.5 (two: M.S. and R.M.A.)
25	Description of the coding tree	Did authors provide a description of the coding tree?	2.5; Table 2; Table S3
26	Derivation of themes	Were themes identified in advance or derived from the data?	2.5 (inductive; CFIR 2.0 applied as a lens in phase five)
27	Software	What software, if applicable, was used to manage the data?	2.5 (QualCoder)
28	Participant checking	Did participants provide feedback on the findings?	2.6 (member reflection with eight participants; changes to Themes 2 and 5)
29	Quotations presented	Were participant quotations presented to illustrate the themes? Was each quotation identified?	3.3–3.8; Table S3 (participant code, role, site)
30	Data and findings consistent	Was there consistency between the data presented and the findings?	3.3–3.8; Table S3
31	Clarity of major themes	Were major themes clearly presented in the findings?	3.2; Table 2
32	Clarity of minor themes	Is there a description of diverse cases or discussion of minor themes?	3.2 (sector-specific variation); 3.3–3.7

Reference: Tong, A.; Sainsbury, P.; Craig, J. Int. J. Qual. Health Care 2007, 19, 349–357. https://doi.org/10.1093/intqhc/mzm042.

File S2. Semi-structured interview guide

The guide was developed by the research team from the opioid stewardship and safe-prescribing literature and the five CFIR 2.0 domains (innovation, outer setting, inner setting, individuals, and implementation process). It was piloted with two clinicians who were not part of the final sample. Core questions were asked of all participants; role-specific questions were added for each professional group. Probes (in italics) were used flexibly. The guide was available in English and Arabic, and participants chose the interview language. Relevant CFIR 2.0 domains are shown in brackets.

Opening (all participants)

Introduction, confirmation of consent and recording, reminder that participants may decline any question, and that hospitals and individuals will not be named. Brief demographic form completed (age, gender, nationality, profession, specialty or unit, years of experience, years at current hospital, highest qualification).

1. **Please describe your role and how you are involved with opioid medicines in your daily work.**
   – *Which patients, units, and opioid products do you deal with most often?*

Section A. Current opioid practices and safe prescribing [Innovation; Individuals]

2. **Talk me through what usually happens, from the decision to start an opioid to the patient's discharge, for a typical patient in your area.**
   – *Who decides the drug, dose, dosage form, and route? Which protocols or order sets are used?*
3. **In your experience, what makes it difficult to prescribe, dispense, or give opioids safely here?**
   – *Can you give a recent example? What happened? What helped or hindered?*

Section B. Dosage forms and routes of administration [Innovation; Inner setting]

4. **How is the route of an opioid (intravenous, intramuscular, oral, transdermal, other) usually chosen for your patients?**
   – *What role do patients' and families' expectations play? Are there situations in which injection is clearly needed?*
5. **When and how are patients switched from parenteral to oral opioids?**
   – *Who notices that a patient is ready? Are there triggers, policies, or delays (nights, weekends)? How does this compare with antibiotics?*
6. **How are conversions between opioids or routes calculated in practice?**
   – *Which equianalgesic table or source is used? How confident are staff? Can you recall a conversion problem?*
7. **What is your experience with higher-risk products such as transdermal fentanyl, extended-release opioids, and patient-controlled analgesia?**
   – *Who starts them, and for whom? What checks or monitoring are in place (e.g., sedation scales, double-checks, capnography)?*
8. **How does the formulary or supply of opioid products affect the choice of dosage form or route?**
   – *Are any oral, liquid, or immediate-release products unavailable or in short supply? What happens then?*
9. **How are opioids handled at discharge?**
   – *How are quantity, tapering, and follow-up decided? What information do patients receive?*

Section C. Organizational structures, data, and governance [Inner setting; Process]

10. **Who in this hospital is responsible for how opioids are used, as distinct from how they are stored and counted?**
    - *Which committees or teams deal with opioids? How do they work together? Is there anything like antimicrobial stewardship for opioids?*
11. **What information does the hospital collect about opioid use and opioid-related harm, and how is it used?**
    - *Are indicators, audits, or feedback reports shared with staff? How are incidents reported?*
12. **How do the electronic prescribing system and the national controlled-drug platform support or hinder safe opioid use?**

Section D. External context, regulation, and accreditation [Outer setting]

13. **How do national regulations, inspections, and accreditation requirements influence opioid practice in your hospital?**
    - *What do inspections and audits focus on? What do they not look at?*

Section E. Specific patient groups [Individuals; Outer setting]

14. **How are patients with sickle cell disease who present with painful crises managed with opioids here?**
    - *How quickly do they receive analgesia? Which routes are used? Are there individualized care plans? How are these patients perceived by staff?*
15. **What happens when staff suspect that a patient has problematic opioid use or a substance use disorder?**
    - *Is there screening, consultation, or referral? What treatment options are available? What are the consequences for the patient?*
16. **How do concerns about addiction, diversion, or complaints affect opioid decisions?**

Section F. Professional roles and workforce [Individuals; Inner setting]

17. **How are physicians, pharmacists, and nurses each involved in opioid decisions, and how well does this work?**
    - *Can pharmacists change or recommend changes to route or dose? How are "as needed" opioid orders interpreted by nurses?*
18. **What training or support have you received on opioid conversion, monitoring, or pain management?**
    - *How do staff turnover and acute pain service coverage affect practice?*

Section G. Improvements and levers [Process; Inner and outer setting]

19. **If you could change three things to make opioid use safer and better in this hospital, what would they be?**
    - *Who would need to be involved? What resources, authority, or policies would be required? What could go wrong?*
20. **What would need to change outside the hospital (e.g., national policy, accreditation, regulation) to support opioid stewardship?**

Role-specific additions

- Physicians: How do you decide between parenteral and oral opioids in emergency, surgical, oncology, or hematology patients? How do you respond to pharmacist recommendations?
- Clinical pharmacists: Which opioid interventions do you make most often? How are they received? What would allow you to contribute more?
- Registered nurses: How do you choose among options in "as needed" orders? How do you monitor sedation? What happens when you think a route change is needed?
- Governance leads: How are opioid issues brought to committees? What data reach leadership? What would a formal stewardship program require?

Closing (all participants)

21. **Is there anything else about opioid use in your hospital that we have not discussed and that you think is important?**

Thank the participant; explain the next steps, including the possible invitation to reflect on preliminary findings.

Table S1. Analytic trail linking illustrative coded excerpts to codes, subthemes, and themes

Excerpts are verbatim extracts reported in the manuscript; codes are the semantic or latent codes attached to them during analysis. The interpretive note summarizes how codes were combined into each subtheme and theme during phases 3–5 of reflexive thematic analysis.

Theme	Subtheme	Illustrative coded excerpt (participant)	Codes	Interpretive note
1. Rules without a steward	1.1 Regulation built for custody, not for optimization	"We are excellent at counting... if a patient stays on IV morphine for five days when he could have taken tablets on day two, nobody will ever ask about it." (P15, clinical pharmacist, H1)	Custody audited closely; appropriateness never examined; compliance consumes attention	Contrasting accounts of rigorous custody and absent appropriateness review across all sites were interpreted as a governance asymmetry rather than a knowledge problem.
	1.2 Stewardship without a mandate	"Everyone owns a small piece... But who owns the whole picture? Honestly, nobody." (P04, anesthesiologist, H3)	Fragmented ownership; committee reacts to incidents; comparison with antimicrobial stewardship	Repeated comparisons with antimicrobial stewardship made the absent mandate visible and anchored the theme name.
	1.3 Data that count but do not inform	"I can tell you exactly how many vials of fentanyl we used last month. I cannot tell you how many patients were over-sedated." (P29, medication safety officer, H1)	Stock data, not use data; under-reporting of harm	Data were interpreted as designed for reconciliation, which links to the absence of stewardship indicators.
2. The needle is the treatment	2.1 A shared belief in the potency of injection	"Tablets do nothing for me, I need the needle... If you give tablets, they think you are refusing to treat him." (P02, emergency physician, H1)	Injection equals real treatment; family pressure; trust built over years (SCD)	Initially coded as patient demand; staff accounts showed the belief was shared, which shaped the final theme name (see member reflection, Section 2.6).
	2.2 Step-down that waits for someone to remember	"The patient is eating a full lunch and I am still giving IV morphine PRN... Tomorrow becomes three days." (P21, registered nurse, H2)	IV continued after oral intake resumed; waiting for the round to switch; pharmacist switch only a recommendation	Absent triggers and authority were interpreted as organizational causes of route inertia.
	2.3 A formulary that shapes the route	"If the only strong oral opioid on the shelf is the long-acting one... Both choices are wrong." (P17, clinical pharmacist, H4)	No immediate-release oral morphine on the shelf; shortages lead to substitution	Separated as a subtheme after member reflection showed supply shaped route independently of beliefs.
3. Calculating in the dark	3.1 No shared equianalgesic language	"I asked three doctors to convert the same patch to oral morphine and got three different answers." (P16, clinical pharmacist, H2)	Multiple tables; phone apps; low confidence outside specialties	Variation was interpreted as a system gap (no institutional table) rather than individual error.
	3.2 High-risk forms without high-reliability systems	"For PCA, the safety is me... If I am busy with five other patients, the safety is less." (P23, registered nurse, H1)	Patch in opioid-naïve patients; PCA depends on individual nurse; variable double-checks	Safety resting on individuals rather than systems linked this subtheme to Themes 1 and 5.
	3.3 Transitions without a plan	"We write 'tramadol for two weeks' because that is what we always write." (P06, general surgeon, H2)	Habitual discharge quantities; no taper; no follow-up	Discharge was interpreted as an unreviewed route and dosage-form decision point.

Theme	Subtheme	Illustrative coded excerpt (participant)	Codes	Interpretive note
4. Caught between two fears	4.1 Fear of addiction and scrutiny	"With tablets for home, if something happens, it is my name on the prescription. So many doctors prefer to keep the patient inside on injections." (P01, emergency physician, H2)	Fear of diversion and legal scrutiny; preference for supervised parenteral doses	Interpreted as a paradox: fear of addiction sustained parenteral use.
	4.2 Fear of under-treatment and complaints	"On one side, the fear of addiction. On the other side, the fear of complaints... each one decides alone." (P10, internal medicine physician, H4)	Complaint pressure; no protective protocol; oscillation	The two fears together named the theme.
	4.3 Labels that follow patients	"From that moment, the patient is not a patient in pain; he is a problem." (P26, registered nurse, H4)	"Drug-seeking" labels; delayed analgesia; erosion of trust (SCD)	Stigma was interpreted as reinforcing insistence on parenteral routes.
	4.4 Stewardship that stops at the hospital door	"There is no consult, no pathway, no medication we can start. So we restrict, and the patient goes to another hospital." (P11, hematologist, H1)	No OUD screening; no addiction consult; no MOUD; restriction instead of treatment	Linked hospital stewardship to the wider SUD prevention agenda.
5. Pharmacists at the door, not at the table	5.1 Recommendations without authority	"By the time my recommendation reaches the team, the decision is already made." (P19, clinical pharmacist, H3)	Advisory role; relationship-dependent influence; absent from rounds	Interpreted as a structural boundary on stewardship capacity.
	5.2 Nurses deciding without a mandate	"So I am choosing the route and the dose, but I have no protocol that tells me how." (P22, registered nurse, H3)	Multi-option "as needed" orders; responsibility without authority	Added after member reflection; broadened the theme from pharmacists to professional boundaries.
	5.3 Workforce turnover and training gaps	"The knowledge walks out of the door with them." (P30, nursing quality director, H2)	Turnover; limited night and weekend acute pain cover; no interprofessional training	Knowledge residing in individuals linked this subtheme back to system design (Themes 1 and 3).

CFIR 2.0 domains for each theme are shown in Table 2 of the manuscript. IV, intravenous; MOUD, medication for opioid use disorder; OUD, opioid use disorder; PCA, patient-controlled analgesia; PRN, as needed; SCD, sickle cell disease; SUD, substance use disorder.
